# A comparison between radiomic biological age and chronological age in estimating kidney function

**DOI:** 10.1038/s41598-025-98297-1

**Published:** 2025-04-18

**Authors:** Radin Alikhani, Steven R. Horbal, Manjunath P. Pai

**Affiliations:** 1 Department of Clinical Pharmacy, College of Pharmacy, University of Michigan, 428 Church St, Ann Arbor, MI 48108 USA; 2 Department of Surgery, University of Michigan, Ann Arbor, MI USA

**Keywords:** Kidney function, Population pharmacokinetics, Aminoglycoside, Biological age, Radiomics, Biomarkers, Translational research

## Abstract

Accurate kidney function estimation hinges on essential markers of age and body size. The relative benefit of both markers has been debated with the emerging biological age and novel body size descriptors. We used radiomic biomarkers of age-related changes in body composition to construct new biological age indices as covariates of kidney function. A retrospective cohort of hospitalized patients with plasma concentrations of aminoglycosides and computed tomography images were evaluated. Aminoglycoside clearance served as a kidney function surrogate. A population pharmacokinetic model was constructed to determine whether biological age indices improved aminoglycoside clearance estimation compared to chronological age. The final dataset included 156 patients (51.92% female) with a median (minimum-maximum) age and body weight of 58 [21, 93] years and 81 [43.8, 139.3] Kg. A 1-compartment clearance model with linear elimination, incorporating biological index, serum creatinine, and drug type best fits the concentration-time data. The best radiomic biological age model included dorsal muscle group area, bone mineral density, visceral fat area, and subcutaneous fat density. The radiomic biological age model offered a modest improvement over the chronological age model. This work offers a proof-of-concept, highlighting the potential for more precise methods for aging-related kidney function estimation to aid personalized pharmacotherapy.

## Introduction

Chronological age (CA) serves as the standard factor used to characterize the interindividual variability of aging in population pharmacokinetic analyses^1^. Recent research suggests biological age better reflects the physiological consequence of aging, and that can be a superior predictor of health outcomes than CA^2^. While CA advances uniformly across all individuals, biological age exhibits variation influenced by numerous insults including physiological and environmental factors^3^. Given this heterogeneity, some people may need to manage multimorbidity and polypharmacy in their 80s, while other peers may not encounter significant health issues, leading an active life without needing regular medical intervention^4,5^. A notable example of this inter-individual variability in organ systems is observed in the kidneys, which progressively deteriorate in conjunction with aging^6^. Kidney function declines after the CA of 40 years^7^. Moving forward, an 80-year-old can have a glomerular filtration rate (GFR) ≥ 90 mL/min or a significantly reduced GFR ≤ 30 mL/min if comorbidities exist.

Recent advancements in omics technologies have driven the development of new tools that can quantitatively characterize biological age^8,9^. One such tool is radiomics, which offers a non-invasive means of measuring biological age^10^. Radiomic-based biomarkers have been used to successfully develop models of brain age and heart age to predict functional outcomes after stroke and cardiovascular events, respectively^11,12^. Radiomics is also capable of providing valuable information about age-related body composition changes^13^. However, clinical pharmacology applications of radiomic-based models of biological age are presently understudied and are sparsely reported in the literature.

Personalized radiomic-based body composition biomarkers offer the potential for a better understanding of human body aging by replacing standard measures of age and body composition in dosing medications^14,15^. To this end, assessing kidney function in older adults is especially important for accurate dosing. Numerous equations for estimating kidney function exist with differing accuracy and precision^7^. As models of kidney function are typically developed as a function of age and body composition, we hypothesized that radiomic-based biomarkers of aging are significant factors in predicting kidney function. To test our hypothesis, we developed biological age indices from Computed Tomography (CT) images and compared them to CA, an essential covariate of aminoglycoside clearance (Cl) through population pharmacokinetic (Pop-PK) analysis. Aminoglycosides undergo routine therapeutic drug monitoring and serve as model compounds for estimating kidney function because their clearance reflects the glomerular filtration rate (GFR). Consequently, this study aims to better understand how radiomic biological age affects kidney function, to improve drug dosing precision.

## Results

Table 1 summarizes the descriptive statistics for patient demographics and the radiomic biomarkers for this study. A total of 156 patients were included in this study, and the percentage of females was slightly higher than that of males (~ 52%). The subjects had a median age [min, max] of 58 [21, 93] years and weight [min, max] of 81 [43.8, 139.3] Kg. Tobramycin was administered to treat 89 patients (57%), and the remainder received gentamicin. Among the significant CT-acquired abdominal biomarkers, psoas expanded muscle area and psoas expanded muscle density measured at the fourth lumbar vertebra (L4) and dorsal muscle group expanded muscle area (dmgexpmarea) measured at L2 were selected due to having the least missing values. The remaining biomarkers were analyzed at their L3 measures.

**Table 1:** Descriptive statistics of the dataset’s patient demographics and morphomic variables.

Characteristic	Data	Range (min, max)
Number of subjects	156	
Age, median (IQR) (Year)	58 (22)	21–93
Gender		
Female (%)	81 (51.92)	
Drug		
Tobramycin (%)	89 (57.05)	
Weight, mean (SD) (Kg)	81.0 (21.06)	43.8–139.3
Height, mean (SD) (cm)	170.5 (10.79)	147.3–203.2
Scr, median (IQR) (mg/dL)	0.75 (0.57)	0.17–5.5
Psoas muscle area at L4, mean (SD) (mm^2^)	1953.7 (748.63)	455.5–4353.5
Psoas muscle density at L4, mean (SD) (HU)	46.28 (11.73)	0.24–72.64
Dorsal muscle group area at L2, mean (SD) (mm^2^)	4030 (1123.49)	1024–7950
Fascial area at L3, mean (SD) (mm^2^)	50,625 (14102.61)	26,787–112,550
Visceral fat area at L3, mean (SD) (mm^2^)	12,790 (10708.68)	40–49,471
Subcutaneous fat density at L3, mean (SD) (mm^2^)	-89.03 (12.76)	−112.08–−59.83
Bone mineral density at L3, mean (SD) (HU)	182.23 (63.03)	46.35–588.30
Vertebral slab height at L3, mean (SD) (mm)	32.78 (3.83)	20.46–42.13

*Scr* serum creatinine, *Clcr* creatinine clearance, *HU* hounsfield unit.

Numerous structural models were investigated to fit the Pop-PK model of aminoglycosides. The model selection process is detailed in the (Supplementary Table 3). A model featuring a 1-compartment structure, including an infusion administration with no delay, linear Cl model, and combined error model (lognormal distribution) showed superior fitting and was set as the base model (AIC = 2617.07). The initial population estimates for the plasma model were 5.6 L/h for Cl and 56.81 L for volume of distribution (V). The best model was determined using AIC, goodness-of-fit, and the residual standard estimates (RSE%) of population parameters.

Three Morphomic Biological Age indices (BA_Mor_1_, BA_Mor_2_, BA_Mor_3_), and Scr were found to exert the most significant influence on Cl and the drug type on the distribution of aminoglycosides. The expansion of the developed BA_Mor_ indices is provided in (Table 2). Scr was incorporated into the models as a denominator of BA_Mor_, which more closely mimicked the Cockcroft-Gault equation and resulted in a lower AIC than adding indices and Scr separately to the model. Table 3 summarizes the final Pop-PK models stratified by age index. Our results demonstrated that BA_Mor_-based Pop-PK models had significantly lower AIC (2607.38, 2611.90, 2609.25) than the CA-based model (2618.49). Also, among the BA_Mor_-based models, the model developed by BA_Mor_3_ exhibited the best fit to the aminoglycoside Cl. Using this index, we could also fit another CT-based biomarker, subcutaneous fat density (subcutfathu_mean), into the aminoglycoside Cl model. The final model developed by BA_Mor_3_ exhibited the lowest AIC, a reduction of approximately 19 points compared to the base model (from 2617.07 to 2598.33).

**Table 2:** Expansion of the models of biological morphomic age.

BA_Mor_1_	($\:Psoas\:muscle\:area*BMD)/Fascia\:area$
BA_Mor_2_	$\:Psoas\:muscle\:area*Psoas\:muscle\:density*Vb\:slab\:height$
BA_Mor_3_	*(* $\:Dorsal\:muscle\:group\:area*BMD)/Visceral\:fat\:area$

*Psoas muscle area* psoas expanded muscle area, *BMD* bone mineral density, *Fascia area* area of fascia, *Psoas muscle density* psoas expanded muscle density, *Vb slab height* vertebra slab height, *Dorsal muscle group area* dorsal muscle group expanded muscle area, *Visceral fat area* visceral adipose tissue area.

**Table 3:** Complete Pop-PK models of aminoglycoside clearance stratified by the type of age in monolix.

	Model	AIC
	Base (1-compartment combined-2)	2617.07
CA	Base + CA/creat_Cl	2618.49
	Base + 1/(CA*creat)_Cl	2609.99
	Base + 1/CA_Cl	2608.62
	Base + 1/(CA*creat)_Cl + drug_V	**2602.12**
BA_Mor_ 1	Base + BA_Mor_1__Cl	2612.44
	Base + BA_Mor_1_/creat_Cl	2607.38
	Base + BA_Mor_1_/creat_Cl + drug_V	**2601.57**
BA_Mor_ 2	Base + BA_Mor_**2**__Cl	2615.93
	Base + BA_Mor_**2**_/creat_Cl	2611.90
	Base + BA_Mor_2_/creat_Cl + drug_V	**2601.71**
BA_Mor_ 3	Base + BA_Mor_**3**__Cl	2613.97
	Base + BA_Mor_3__Cl + subcutfathu_Cl	2609.01
	Base + BA_Mor_3_/creat_Cl + subcutfathu_Cl	2609.25
	Base + BA_Mor_3_/creat_Cl + subcutfathu_Cl + drug_V	**2598.33**

^1^BA_Mor_1_: $$\:\frac{Psoasexpmuscarea\:*BMD}{Fasciaarea}$$.

^2^BA_Mor_2_: $$\:Psoasexpmuscarea*PsoasexpmuscHU*Vbslabheight$$.

^3^BA_Mor_3_: $$\:\frac{DMGexpmarea\:*BMD}{VATarea}$$.

^4^subcutfathu: subcutaneous fat density.

The values in bold represent models with the lowest AIC value by age category.

**Fig. 1:**
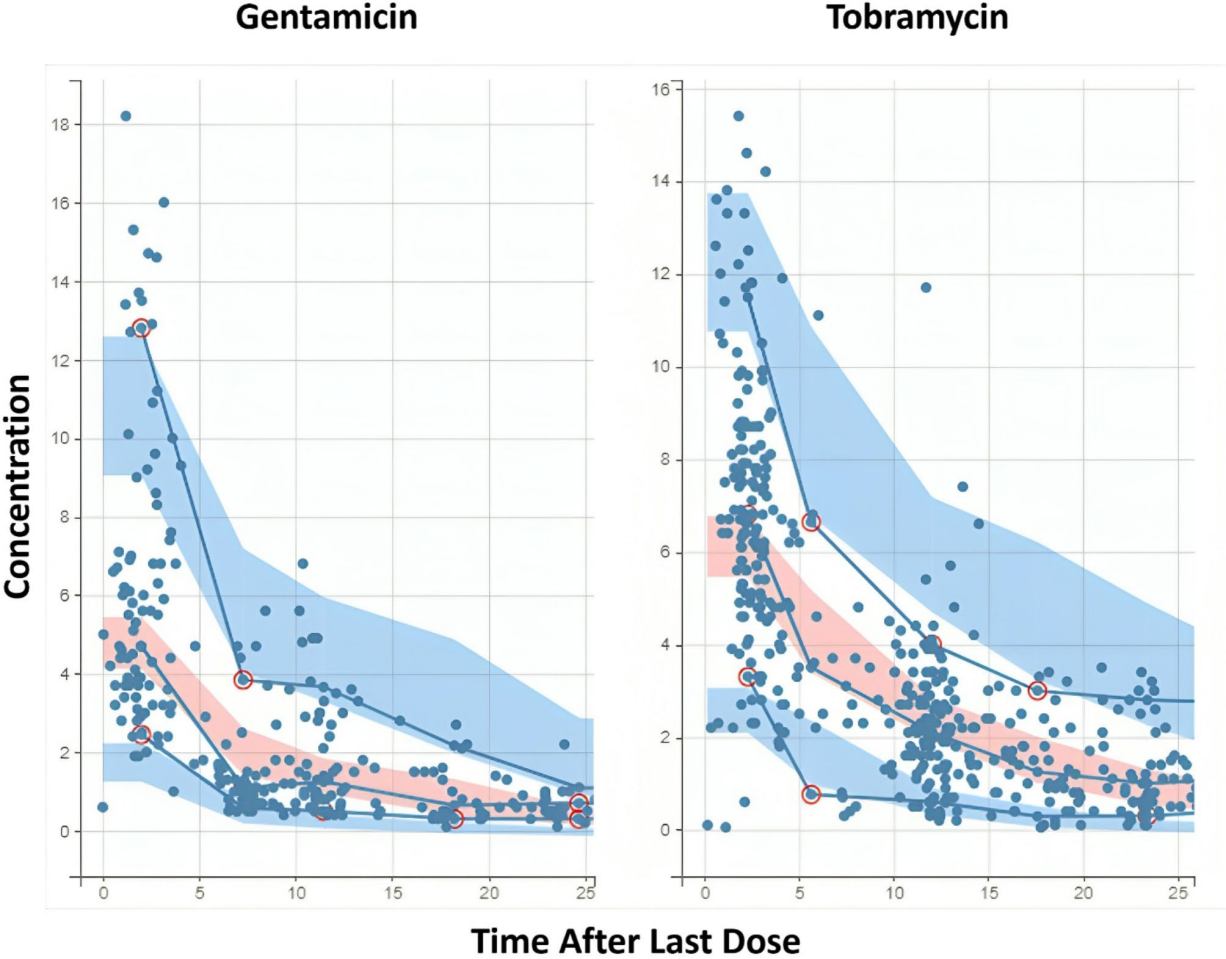
Visual predictive check (VPC) plot for the final aminoglycoside clearance model stratified by drug type. The observed data are depicted as dots, with prediction intervals shown as shaded areas. Red circles highlight observed data points that deviate from the predicted range, suggesting potential outliers.

A summary of the population parameter estimates for the final model of each age type is provided in (Supplementary Table 4). The population estimates for Cl and V in the best model were 2.62 L/h and 45.89 L, respectively. The predicted aminoglycoside Cl value in the best model was comparably lower than that with BA_Mor_1_, BA_Mor_2_, and CA (2.62 L/h vs. 5.80, 5.72, and 5.82 L/h). The power of BA_Mor_ indices for Cl (β_Cl_BA_Mor_) was slightly lower than the power of CA for Cl (β_Cl_CA = 0.19) in their second decimal; however, the power of subcutaneous fat density in the final model was significantly greater than the CA (β_Cl_subcutfathu= −0.93). The fixed effects for the volume of distribution stratified by drug type in BA_Mor_-based models were generally lower than the CA-based model, with BA_Mor_3_ having the lowest value for both drugs (45.89 L for gentamicin and 60.75 L for tobramycin) and BA_Mor_1_ having the lowest RSE% (6.66 for gentamicin and 5.17 for tobramycin) among other types of age. The standard deviation (SD) of the random effects associated with V and Cl (Ω_V and Ω_Cl) were relatively similar across the four age types, with BA_Mor_3_ having a slightly lower Ω_V which indicates less variability among individuals. Regarding the error model parameters α and β, BA_Mor_1_ exhibited a lower α than CA, while BA_Mor_2_ and BA_Mor_3_ demonstrated a lower β than CA. Figure 1 represents the visual predictive check (VPC) plot for the best aminoglycoside Cl model. The final Pop-PK model equation is as follows:

log (Cl) (L/h) = 0.42 + 0.12 × log ($\:\frac{BAMor\_3}{Scr*300}$) – 0.92 × $\:\frac{SubcutfatHU}{90}$ + exp (ηCl) (1)

In the next step, we removed Scr and drug type from the models to compare the effect of only CA and BA_Mor_ alone on aminoglycoside Cl. As expected, CA was significant in predicting aminoglycoside Cl. The results for BA_Mor_ indices revealed that BA_Mor_1_ and BA_Mor_3_ remained significant in predicting aminoglycoside clearance even after withholding other covariates. However, BA_Mor_2_ lost its significance in the absence of other covariates. The CA-only Pop-PK model showed a comparably lower AIC than each BA_Mor_ index alone (2608.62 vs. 2612.44, 2615.93, and 2613.97). The model fit with CA was better than BA_Mor_2_, equal to BA_Mor_1_, and weaker than BA_Mor_3_. Although the age indices were found significant in the BA_Mor_1_- or CA-only Pop-PK models, each model was considered incomplete in Monolix^®^ (version 2024R1; Lixoft, Antony, France), and required additional covariates such as drug type under volume of distribution, to be complete. Yet, we could successfully model a Pop-PK model for aminoglycoside Cl using *only* BA_Mor_3_ and subcutaneous fat density biomarkers. Table 3 provides the AIC of the three Pop-PK models for each age index: (1) Pop-PK model with an age index only; (2) Pop-PK model with CA or BA_Mor_ to creatinine ratio; (3) Pop-PK model with the ratio in model 2 and drug type under volume of distribution.

The summarized population parameter estimates for the age-only models after stratifying by age are provided in (Supplementary Table 5). It reveals that the population estimates for volume were similar across the ages, whereas the model with BA_Mor_3_ remained the model with the lowest predicted Cl (2.62 L/h). After removing the effect of other covariates, the power of BA_Mor_ and CA indices for Cl (β_Cl_BA_Mor_ and β_Cl_CA) persisted almost unchanged, except for BA_Mor__2, which was notably dropped (0.12 vs. 0.089). Similar to the final models for each age index, the SD of the random effects associated with V and Cl (Ω_V and Ω_Cl) were pretty similar across the four age types, and no meaningful association was found. Furthermore, although the error model parameter α for BA_Mor_2_ and BA_Mor_3_ were reduced, the RSE% of this parameter was increased in both indices.

## Discussion

**Fig. 2:**
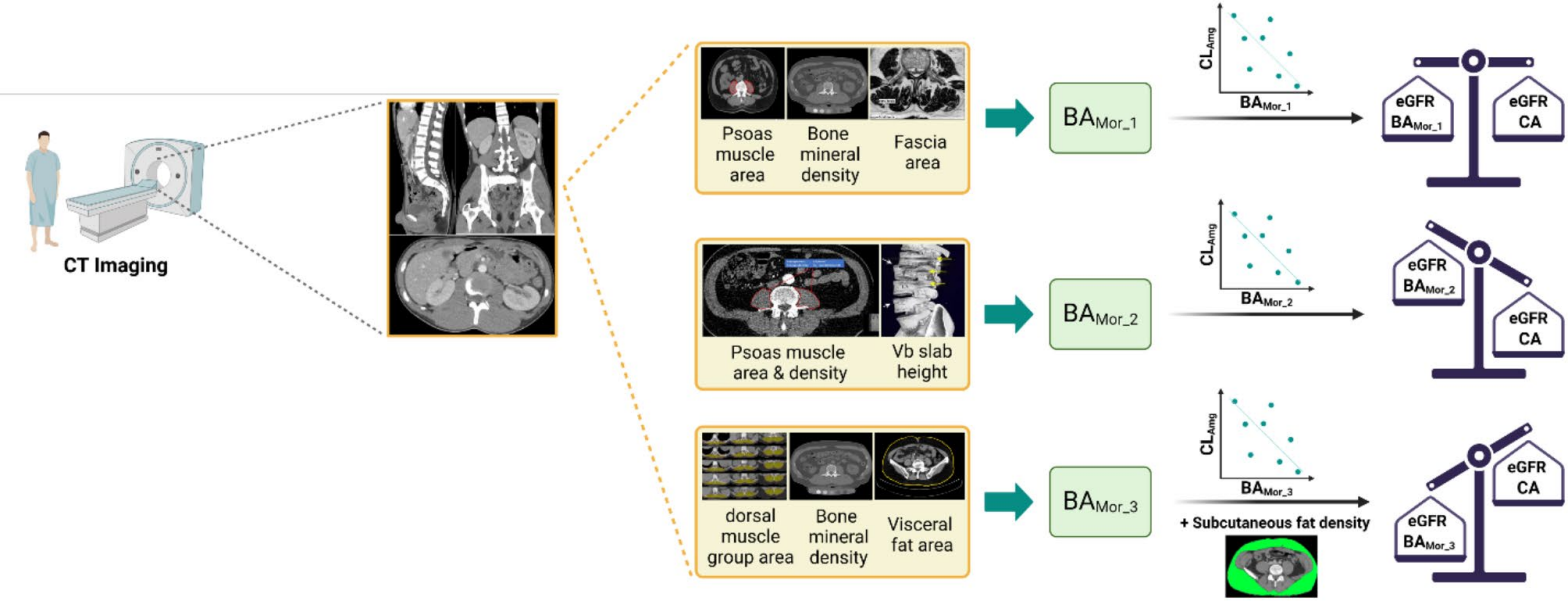
Schematic of comparing the effectiveness of three proposed BA_Mor_ with chronological age in predicting kidney function. (Made with BioRender.com).

This study’s objective was to identify improved predictors of kidney function to replace age and body composition. We examined how our new BA_Mor_ indices compare with CA in improving the precision of estimating kidney function in Pop-PK models. Our findings were mixed with the application of BA as a potential alternative to CA to construct models that can improve the precision of estimating kidney function (Fig. 2). Indices containing age-related biomarkers for major body composition components including muscle, bone, and fat (BA_Mor_1_ and BA_Mor_3_) performed better than an index for muscle mass to reflect creatinine production by human muscles (BA_Mor_2_). A concise overview of the individual elements that make up each of the BA_Mor_ indices are discussed as follows.

The kidney’s long axis runs parallel to the lateral edge of the psoas muscle and is positioned in front of the quadratus lumborum muscle, a type of dorsal muscle situated deep in the lower back, adjacent to the intrinsic muscles of the spine^16,17^. Due to this parallel alignment with the psoas muscle, the kidneys are oriented at an oblique angle, with the superior pole being more medial and posterior compared to the inferior pole^17^. This distinctive position supports the correlation between kidney function and BA_Mor_1_ and BA_Mor_2_, which incorporated psoas muscle area and/or psoas muscle density in themselves, and BA_Mor_3_, which involved dorsal muscle area.

BA_Mor_1_ integrates the psoas muscle area, bone mineral density (BMD), and fascia area. The psoas muscle is previously validated as a key indicator of overall muscle health and aminoglycoside Cl^18,19^. The BMD component reflects bone strength, which typically decreases with age^20^. It can also be associated with the vitamin D_3_ activating function of kidneys indirectly, resulting in a faster loss of bone mass^20^. The fascia area acts as a structural component, influencing the functionality of muscles, tissues, and organs (i.e. kidneys)^21^. This index, therefore, provides a composite measure that combines aspects of body composition changes with increasing age, related to kidney function. The resulting measure seems to match CA in kidney function estimation.

BA_Mor_2_ is a composite index of muscle mass, muscle tissue quality, and skeletal health. Other than the psoas muscle area discussed earlier, this index involves psoas muscle density, which reflects the quality of muscle tissue, potentially indicating fat infiltration and overall muscle conditioning, as well as vertebral slab height, an indicator of cumulative life course biomechanical strain, that could be linked to kidneys as the kidney length should be between three and four vertebral body lengths^22,23^. As this index was primarily intended to represent muscle mass and creatinine production, the poor performance was surprising when other covariates were withheld, and suggests that although the index may seem fundamentally intuitive, combining these biomarkers might introduce variability that doesn’t improve the prediction of kidney function as effectively.

BA_Mor_3_ combines the area of the dorsal muscle group with BMD, normalized by the visceral fat area. Combining the dorsal muscle area with BMD provides a robust indicator of musculoskeletal integrity, as dorsal muscles are crucial for posture and overall musculoskeletal health when it comes to complications such as muscle fatigue, back pain, and spinal dysfunction^24,25^. Normalizing by visceral fat area adjusts for metabolic health, as high visceral fat can be associated with obesity and metabolic syndrome^26^. Most importantly, increased visceral fat accumulation leads to the accumulation of fat around the kidneys and within the renal sinus, which can ultimately result in increased intrarenal pressure, reduced renal blood flow, greater sodium reabsorption, glomerular hyperfiltration, and kidney dysfunction^27^. Thus, a high amount of visceral fat is a crucial indicator of kidney prognosis in patients with CKD^28,29^. BA_Mor_3_’s outperformance in our models suggests that this index captures a more holistic view of biological aging in body composition that better correlates with kidney function than CA or other BA_Mor_ indices.

While these findings are promising, the BA-structured models performed similarly to the CA-structured model. While this study only focused on the radiomic biological age, alternate composite measures of BA should be tested for their significance with aging in the future. A more precise measurement of aging could improve kidney function estimation on the individual level, which is required for precision dosing and staging chronic kidney disease. Our findings underscore composite age-informed radiomic biomarkers encompassing body composition from various dimensions to improve kidney function estimates better than CA. These findings are particularly important for populations requiring more precise dosing, such as older adults, patients with obesity, and patients with impaired kidney function, as radiomic biomarkers provide a nuanced and personalized assessment of changes in body composition with aging. Our results provide initial evidence, yet further positive evidence is needed to support the clinical implementation of radiomics in biologically informed drug dosing, particularly given existing barriers such as cost and the complexity of data analysis. Future research is encouraged to validate our findings in the target population and incorporate other biomarkers such as kidney-specific imaging metrics or epigenetic factors to refine the BA indices. Also, longitudinal studies assessing changes in BA indices over time and their correlation with kidney function decline could provide deeper insights into the kidney function changes with biological aging.

This study is, to our knowledge, the first to apply radiomic-based BA for a clinical pharmacology application. However, it has some limitations. First, this work is cross-sectional, and results cannot indicate a causal relationship. Second, the study has hospital-based selection bias (Berkson’s bias) and may not be generalizable to other populations. Third, the sample size is small, consisting of only 156 patients. Future studies with larger sample sizes are needed to validate the robustness of these proposed indices. Next, the biological indices were primarily developed using measurements at the L3 vertebra level to simplify the analysis and overlooked potentially significant measurements at other vertebral levels. Next, we did not have access to any kidney-specific CT biomarkers, such as kidney parenchyma volume. We relied heavily on biomarkers representing major body composition components instead of kidney-specific imaging biomarkers. Finally, we used only Scr as the endogenous kidney biomarker and could not incorporate serum cystatin C into our models because it was unavailable for our patients. Regardless, this work provides evidence to support the use of radiomics in pharmacological investigations for stronger estimation of interindividual variability as well as control of related statistical confounding.

In conclusion, medical imaging techniques provide valuable insights into phenotypic and unseen changes in body composition. This information can be aggregated into the models of biological aging, which can be correlated with clinical outcomes such as kidney function and chronic kidney disease staging. We successfully used CT scan imaging data to develop BA_Mor_ indices in the Pop-PK models of aminoglycoside Cl as a surrogate for kidney function.

## Materials and methods

Data from 363 adult participants with available CT scan images were retrieved retrospectively from the medical record and the picture archive and communication system (PACS) at the University of Michigan Health System. Approval from the internal review board (IRB) was secured before starting the study (HUM00041441). Informed consent was waived by the IRB and all methods were performed in accordance with the relevant guidelines and regulations, including the Declaration of Helsinki. Inclusion was restricted to patients with at least three doses and concentrations of aminoglycoside whose IDs matched the aminoglycoside PK data. Patients who underwent treatment with Amikacin were excluded. The final cohort consisted of 156 subjects, having several CT-based biomarkers (*n* = 28) measured at different vertebra numbers. A complete list of these biomarkers along with their definition and vertebra number is provided in (Supplementary Tables 1, 2).

Analyses were primarily restricted to measurements at L3 due to its prevalent use in clinical research. CT scans measuring the cross-sectional skeletal muscle area at L3 are commonly used to evaluate sarcopenic low muscle mass^30,31^. Since other vertebral levels can correlate with L3, measurements from neighbor lumbar vertebrae (L2 or L4) were used as a proxy in cases of unavailable L3 measures for biomarkers (Supplementary Table 1). Some of these biomarkers contained missing values in a few patients, which were handled using the mice package v3.16.0 with the random forest method for data imputation in R Statistical Software (v4.4.1; R Core Team 2024; http://www.R-project.org)^32^.

Aminoglycoside plasma concentrations were analyzed using nonlinear mixed-effects modeling with the stochastic approximation expectation minimization (SAEM) algorithm in Monolix^®^. Model development employed a multistage approach based on a 1-compartment PK model. Initially, a linear model for infusion administration with no delay parameterized with V and Cl was tested. The log-normal distribution was adopted for all individual parameters. Various error models (constant, proportional, or combined) were evaluated to assess residual variability.

To achieve this project’s goal, various combinations of CT-based biomarkers were developed as a BA_Mor_ index and tested against the aminoglycoside Cl in Pop-PK models to improve kidney function. The biomarkers were either identified as significant in Monolix^®^ or established as a marker of body composition in previous studies. After evaluating different combinations, we selected three BA_Mor_ indices (BA_Mor_1_, BA_Mor_2_, BA_Mor_3_), as shown in (Table 2). Along with these indices, the effects of covariates such as age, sex, drug type, serum creatinine (Scr), and CT-based biomarkers on aminoglycoside Cl were investigated. Considering kidney function is inversely correlated with age, both CA and inverted CA (1/CA) were tested on aminoglycoside Cl^33^. Each covariate’s effect was tested and determined whether they should be added to the model by Pearson’s correlation to the between-subject variability of pharmacokinetic parameters. Model discrimination was based on an at least two-point reduction in the Akaike information criterion (AIC) between models. Model performance was assessed by evaluating the goodness of fit between observed and predicted concentrations, ensuring that the pharmacokinetic parameters exhibited a low relative standard error, and examining the distribution of individual weighted residuals (IWRES) and normalized prediction distribution errors (NPDE). These metrics were collectively used to gauge the model’s accuracy, precision, and predictive reliability.

## Electronic supplementary material

Below is the link to the electronic supplementary material.

Supplementary Material 1

## Data Availability

All data supporting the findings of this study are available within the paper and its Supplementary Information. All data are available from the corresponding author upon reasonable request.
