# Supplementary material for: A comparison between radiomic biological age and chronological age in estimating kidney function
Source: Sci Rep. 2025 Apr 18;15:13384. doi: 10.1038/s41598-025-98297-1 (PMC12008189; doi:10.1038/s41598-025-98297-1)
Supplement: Supplementary file 1 — Supplementary Material 1 [file 41598_2025_98297_MOESM1_ESM.docx]

A Comparison Between Radiomic Biological Age and Chronological Age
in Estimating Kidney Function **(Supplementary File)**

**Supplementary Table 1** – List of the morphomic variables and their pragmatic means in the final cohort (n=156)

| Morphomic variable | Selected vertebra no. | Mean |
| --- | --- | --- |
| Mean_ptsonborder_skin | L3 | 0.11761 |
| Vbslabheight | L3 | 32.78 |
| Bmdhuvbaligned | L3 | 182.23 |
| Fascia2frontskin | L3 | 19.809 |
| Vb2fascia | L3 | 114.87 |
| Sp2backskin | L3 | 31.958 |
| Bodydepth | L3 | 260.2 |
| Fasciadepth | L3 | 213.6 |
| Fasciaarea | L3 | 50625 |
| Visceralfatarea | L3 | 12790 |
| Visceralfathu_mean | L3 | -86.70 |
| Subcutfatarea | L3 | 18731.42 |
| Subcutfathu_mean | L3 | -89.03 |
| Dmgexpmarea | L2 | 4030 |
| Dmgndmarea | L2 | 2470.6 |
| Dmgldmarea | L2 | 979.4 |
| Dmgexpmmeanhu | L2 | 38.651 |
| Expmuscarea | L3 | 13185 |
| Hdmarea | L3 | 242.31 |
| Ndmarea | L3 | 7517.9 |
| Ldmarea | L3 | 3638.0 |
| Vldmarea | L3 | 1791.2 |
| Expmuscmeanhu | L3 | 34.6695 |
| Total_psoas_expmarea | L4 | 1953.7 |
| Total_psoas_ndmarea | L4 | 1453.17 |
| Total_psoas_ldmarea | L4 | 367.665 |
| Avg_psoas_expmmeanhu | L4 | 46.2854 |
| Wallpcnt_calc | L3 | 14.661 |

**Supplementary Table 2** – List of Potential Morphomic Parameters Extracted by Vertebral Level

| **Body Measures** | |
| --- | --- |
| bodydepth | Front-to-back body distance (aligned to body habitus) (mm) |
| bodywidth | Left-to-right body distance (aligned to body habitus) (mm) |
| vbslabheight | Height of body slab at the vertebra (mm) |
| mean_ptsonborder_skin | Average number of points on the skin border detected during image segmentation. |
| sp2backskin | Distance - posterior tip of spinous process to back skin (central back fat depth) (mm) |
| vb2fascia | Distance - anterior vertebral body to prevertebral fascia  (mm) |
| fascia2frontskin | Linear distance from anterior fascia to anterior skin (central sub-cutaneous depth) (mm) |
| fasciadepth | thickness or depth of the fascial layer from the superficial to deep fascia (mm) |
| **Muscle (skeletal) Measures** | |
| expmuscarea | Cross sectional area of the muscle pixels (-29 to 150 HU) between the muscle wall and fascia boundaries (excluding filled bone, spinal canal, and disk) (mm^2^) |
| expmuscmeanhu | Mean pixel intensity of the skeletal muscle pixels (-29 to 150 HU) |
| vldmarea | Cross sectional area of the skeletal muscle falling in a very low density muscle HU range (-29 to -1) (mm^2^) |
| ldmarea | Cross sectional area of the skeletal muscle falling in a low density muscle HU range (0 to 30) (mm^2^) |
| ndmarea | Cross sectional area of the skeletal muscle falling in a normal density muscle HU range (30 to 100) (mm^2^) |
| hdmarea | Cross sectional area of the skeletal muscle falling in a high-density muscle HU range (100 to 150) (mm^2^) |
| **Muscle (Psoas) Measures** | |
| Total_psoas_expmarea | (PMA) Cross sectional area of the muscle pixels (-29 to 150 HU, expanded range) within the perimeter of the left and right psoas. (mm^2^) |
| Total_psoas_ndmarea | Cross sectional area of the psoas falling in a normal density muscle HU range (31 to 100) (mm^2^) |
| Total_psoas_ldmarea | Cross sectional area of the psoas falling in a low-density muscle HU range (0 to 30) (mm^2^) |
| Avg_psoas_expmmeanhu | Mean pixel intensity of all pixels inside the total psoas area. (HU) |
| **Muscle (Dorsal muscle group) Measures** | |
| Dmgexpmarea | Cross sectional area of the muscle pixels (-29 to 150 HU, expanded range) within the DMG perimeter. (mm^2^) |
| Dmgndmarea | Cross sectional area of the DMG falling in a normal density muscle HU range (31 to 100) (mm^2^) |
| Dmgldmarea | Cross sectional area of the DMG falling in a low density muscle HU range (0 to 30) (mm^2^) |
| Dmgexpmmeanhu | Mean pixel intensity of muscle pixels (-29 to 150 HU) within the DMG boundary. (HU) |
| **Fat Measures** | |
| subcutfatarea | Area between skin and fascia meeting fat density thresholds (-205 to -51 HU) (mm2) |
| visceralfatarea | Area inside fascia meeting fat density thresholds (-205 to -51 HU) (mm2) |
| subcutfathu | Median pixel intensity of fat-intensity pixels (-205 to -51 HU) in the subcutaneous region. |
| visceralfathu | Median pixel intensity of fat-intensity pixels (-205 to -51HU) inside the visceral cavity. |
| **Spine (trabecular bone) Measures** | |
| bmdhuvbaligned | Average pixel intensity (in CT Hounsfield Units) inside central bone core sample (HU) |
| **Miscellaneous** | |
| wallpcnt_calc | Calculated percentage of the abdominal aortic wall that is occupied by calcification (%) |

**Supplementary Table 3** – Summary of key models leading to selection of the final model, reported based off of AIC and software proposal.

| **No.** | **Model expansion** | **AIC** | **Findings** | **Note** |
| --- | --- | --- | --- | --- |
| 1 | Base(constant) | 3117 |  |  |
| 2 | Base(proportional) | 2622.66 |  |  |
| 3 | Base(combined1) | 2622.74 |  |  |
| 4 | Base(combined2) | 2617.07 | Best model | Set as base model |
|  | | | | |
| 5 | Base + log(Age/50)_CL | 2608.62 | - Significant reduction in AIC  - Beta_Cl_logAge: -0.39  Proposal: add drug_V |  |
| 6 | Base + log(1/(Age*creat)/0.03)_CL | 2609.99 | - Beta_Cl_lndex: 0.19  - Proposal: **keep** it and add psoas_expmuscarea to CL | Can we find any morphomic covariate with comparable AIC? |
| 6.1 | Base + log(1/(Age*creat)/0.03)_CL + drug_V | 2602.12 | - Proposal: **keep** it and add psoas_expmuscarea to CL | Current model is NOT complete |
|  | | | | |
| 7 | Base + log((psoasexparea2fasciaarea)/0.04)_CL | 2614.52 | - AIC worsened  - Beta_Cl_lndex: 0.21  - Proposal: Model6+psoas |  |
| 8 | Base + log(psoasexpmuscarea/2000)_CL | 2616.54 | - Beta_Cl_lndex: 0.18  - Proposal: Model6+psoas |  |
| 9 | Base + log(psoasexpmuscarea2creat/2700)_CL | 2609.48 | - Beta_Cl_lndex: 0.23  - **RSE for “a” NOT intuitive**  - Proposal: **keep** the model and add Drug_V | Higher strength beta_Cl_lndex: 0.23 |
| 10 | Base + log(dmgexpmuscarea2creat/5500)_CL | 2611.64 | - Beta_Cl_lndex: 0.21  - Parameter estimates good  Proposal: **remove** index, add bmd_CL and drug_V_CL |  |
| 10.1 | Base + log(dmgexpmuscarea/3800)_CL | 2624.06 | **Proposal: remove** dmg, add psoasexpmuscarea and lo | Not a good index alone |
| 11 | Base + log(bmd2creat/250)_CL | 2610.2 | - Beta_Cl_lndex: 0.18  - Proposal: **keep** it and add drug_V |  |
| 11.1 | Base + log(bmd/180)_CL | 2612.12 | - Beta_Cl_lndex: 0.29  Proposal: **remove** the index, add bmd_CL and drug_V_CL | Weight of bmd is pretty high |
| 11.2 | Base + log(bmd/180)_CL + log(Age/50)_CL | 2609.23 | Proposal: Proposal: **remove** bmd and keep age, add drug_V_CL | Weight of bmd decreases in the presence of age |
| 12 | Base + log(expmuscarea2creat/18300)_CL | 2613.05 | - AIC worsened  - Proposal: **remove** index and add logAge_CL | Higher strength beta_Cl_lndex: 0.22 |
| 13 | Base + (wallpcnt_calc2creat)/20_CL | 2618.12 | - AIC worsened, beta reduced dramatically  - Proposal: **remove** index, add logAge_CL and drug_V | Could not do log-transform for wallpcnt_calc |
| 14 | Base + log(psoasexpmuscarea / psoasexphu2creat/85)_CL | 2615.88 | - AIC worsened  - Proposal: **remove** index, add logAge_CL and drug_V | Muscle area to density not working |
| 15 | Base + log(psoasexparea*bmd)2creat/555200)_CL | 2607.08 | - Lowest AIC so far  - Beta_Cl_lndex: 0.18  - parameter a RSE: 74%  - Proposal: **keep** it w/o adding anything else | Good index. |
| 16 | Base + log(psoasexparea*bmd)/VATarea 2creat/320)_CL | 2612.62 | - AIC increased  - Beta_Cl_lndex: 0.074  - Proposal: **keep** the index and add SubQhu_CL | Adding SubQ is interesting and a good sign, hopefully be able to add a better fat index to the model  * also try VAT/SAT |
| 17 | Base + log(psoasexparea*bmd)/VATarea 2creat/320)_CL + subQhu/90_CL | 2607.04 | Proposal: **keep** the model | Actually, very similar to model 15 |
| 18 | Base + (psoasexparea*bmd)/ (VATarea*subQhu)2creat/5)_CL | 2618.2 | Proposal: **remove** the index, add bmd_CL and logAge_V | Could not log-transform subQhu, so removed log for the entire index but it didn’t work. |
| 19 | Base + log(psoasexparea*bmd)/Fasciaarea 2creat/10)_CL | 2607.38 | - AIC good  - Beta_Cl_lndex: 0.16  - Proposal: **keep** the index and add drug_V |  |
| 19.1 | Base + log(psoasexparea*bmd)/Fasciaarea 2creat/10)_CL + drug_V | 2601.57 | Model fit is pretty good!  Proposal: **Keep the model** | One potential final model |
| 20 | Base + log(psoasexparea)/Fasciaarea 2creat/0.05)_CL | 2610.85 | - Parameter estimates pretty good  - Beta_Cl_lndex: 0.19  - Proposal: **keep** the model | Bmd improves AIC, but reduces beta |
| 21 | Base + log(expmuscarea*bmd)/Fasciaarea 2creat/80)_CL | 2609.35 | - parameter a RSE: 109%  - Beta_Cl_lndex: 0.16  - Proposal: **keep** the index and add logAge_CL | Psoasexpmusc is better than expmsuc |
| 22 | Base + log(expmuscarea*bmd2creat/3580000)_CL | 2608.11 | - Beta_Cl_lndex: 0.19  - Proposal: **keep** the index and add drug_V |  |
| 23 | Base + log(expmuscarea/fasciaarea2creat/0.4)_CL | 2612.35 | Proposal: **remove** the index and add logAge_CL | This index is not working |
| 24 | Base + log(VAT/SAT2creat)_CL | 2618.17 | Proposal: **remove** the index, add bmd_CL and drug_V_CL | VAT/SAT is an important morphomic index but it is not working here. |
| 25 | Base + log(psoasexpmuscarea*psoasexphu *vbslabheight2creat/4200000) | 2611.9 | Proposal: **Keep** the index and add drug_V  Beta_Cl_index: 0.13 |  |
| 25.1 | Base + log(psoasexpmuscarea*psoasexphu *vbslabheight2creat/4200000) + drug_V | 2601.71 | Model fit is pretty good!  Proposal: **Keep the model** | Another potential final model |
| 26 | Base + log(weight2creat/120) | 2616.54 | Proposal: **remove** the index and add bmd_CL, drug_CL and logAge_V | At least, morphomics works better than weight. |
| **Additional morphomics ratio from articles** | | | | |
| 27 | Base + log(psoasexparea*bmd)/Fasciaarea *fasciadepth)2creat/10)_CL | 2609.02 | Proposal: keep the model as it is.  - **RSE for “a”: 82** |  |
| 28 | Base + log(dmgld/dmgnd/creat) | 2618.25 | - Not a good model  - Proposal: remove index, add logAge_V_CL | Found this ratio at UM morphomics website |
| 29 | Base + log(dmgexp*bmd/VATarea/creat)/580) | 2612.38 | - Parameter estimates good  - Proposal: keep index, add subQhu/90_CL and drug_V |  |
| 29.1 | Base + log(dmgexp*bmd/VATarea/creat)/580) + subQhu/90 | 2609.25 | - Proposal: keep the model  - Drug_V significant |  |
| 29.2 | Base + log(dmgexp*bmd/VATarea/creat)/580) + subQhu/90 | 2598.33 | - Lowest AIC  - Proposal: keep the model  - No other significant covariates | Another potential final model |
| **Modeling morphomic indexes ONLY** | | | | |
| 30 | Base+log(morphomic_index1)_CL | 2612.44 | Higher AIC than Age  Proposal: **keep** it and add age_V | Parameter estimate pretty good |
| 31 | Base+log(morphomic_index2/3000000)_CL | 2615.93 | Proposal: **remove** it, add 1/(age*creat)_CL, drug_V and psoasexparea_CL |  |
| 32 | Base+log(morphomic_index3)_CL | 2613.97 | Proposal: keep it and add subQhu_CL |  |
| 32.1 | Base+log(morphomic_index3)_CL+ subQhu_CL | 2609.01 | Proposal: keep the model as it is  Beta_CL_index: 0.12  Beta_CL_subQhu: -0.92 |  |
| 33 | Base+log(psoasarea.bmd/300000)_CL | 2612.69 | Beta_CL_index: 0.17  Proposal: keep it and add 1/(age*creat)_CL |  |
| 34 | Base+log(age/creat/80)_CL | 2618.49 | - Beta_CL_index: -0.016  - RSE for index is insane!  - Proposal: remove index, add bmd_CL, drug_CL, age_V |  |
| 34.1 | Base+log(age/creat/80)_CL + drug_V | 2608.97 | Proposal: remove index and add bmd_CL |  |

**Supplementary Table 4** – Final population PK model parameters for the aminoglycosides stratified by age index.

| **Model number** | 19.1  BA_Mor_1_-based | | 25.1  BA_Mor_2_-based | | 29.2  BA_Mor_3_-based | | 6.1  CA-based | |
| --- | --- | --- | --- | --- | --- | --- | --- | --- |
|  | Value  (SE) | RSE% | Value  (SE) | RSE% | Value  (SE) | RSE% | Value  (SE) | RSE% |
| **Fixed effects** | | | | | | | | |
| V_pop | 49.09  (3.27) | 6.66 | 45.87 (3.39) | 7.39 | 45.89 (3.23) | 7.03 | 49.2 (3.46) | 7.04 |
| Beta_V_drug_tobramycin | 0.24  (0.083) | 34.5 | 0.29 (0.087) | 29.5 | 0.28 (0.085) | 30.2 | 0.25 (0.086) | 33.8 |
| Cl_pop | 5.8 (0.26) | 4.48 | 5.72 (0.27) | 4.65 | 2.62 (1.02) | 38.9 | 5.82 (0.26) | 4.55 |
| Beta_Cl_(BA_Mor_/creat) | 0.15 (0.047) | 30.5 | 0.12 (0.046) | 38.2 | 0.11 | 29.4 |  |  |
| Beta_Cl_(subcutfathu) |  |  |  |  | -0.93 (0.38) | 40.5 |  |  |
| Beta_Cl_(CA^-1^/creat) |  |  |  |  |  |  | 0.19 (0.062) | 32.7 |
| **Fixed effects by category** | | | | | | | | |
| V_drug_Gentamicin | 49.09 (3.27) | 6.66 | 45.87 (3.39) | 7.39 | 45.89 (3.23) | 7.03 | 49.2 (3.46) | 7.04 |
| V_drug_Tobramycin | 62.38 (3.23) | 5.17 | 61.61 (3.21) | 5.21 | 60.75 (3.22) | 5.31 | 63.42 (3.34) | 5.26 |
| **Standard deviation of the random effects** | | | | | | | | |
| Omega_V | 0.37 (0.038) | 10.5 | 0.37 (0.043) | 11.6 | 0.36 (0.039) | 10.7 | 0.38 (0.04) | 10.7 |
| Omega_Cl | 0.49 (52.63) | 6.43 | 0.51 (0.034) | 6.61 | 0.49 (0.032) | 6.53 | 0.49 (0.032) | 6.41 |
| **Error model parameters** | | | | | | | | |
| a | 0.049 (0.023) | 46.7 | 0.12 (0.043) | 34.9 | 0.13 (0.038) | 30.5 | 0.069 (0.037) | 53.5 |
| b | 0.4 (0.013) | 3.38 | 0.38 (0.014) | 3.59 | 0.39 (0.014) | 3.67 | 0.4 (0.014) | 3.51 |

**Supplementary Table 5** – Final population PK model parameters for the aminoglycosides built upon age type ONLY.

| **Model number** | 30  (BA_Mor_1_) | | 31  (BA_Mor_2_) | | 32.1  (BA_Mor_3_) | | 5  (CA) | |
| --- | --- | --- | --- | --- | --- | --- | --- | --- |
|  | Value  (SE) | RSE% | Value  (SE) | RSE% | Value  (SE) | RSE% | Value  (SE) | RSE% |
| **Fixed effects** | | | | | | | | |
| V_pop | 56.75 (2.52) | 4.45 | 56.75 (2.7) | 4.75 | 56.11 (2.56) | 4.57 | 56.28 (2.44) | 4.33 |
| Cl_pop | 4.18 (0.48) | 11.5 | 5.69 (0.27) | 4.66 | 2.62 (1.04) | 39.8 | 5.69 (0.25) | 4.38 |
| Beta_cl_(BA_Mor_/creat) | 0.16 (0.059) | 36.6 | 0.089 (0.051) | 57.6 | 0.12 (0.038) | 31 |  |  |
| Beta_Cl_(subcutfathu) |  |  |  |  | -0.92 (0.38) | 41.6 |  |  |
| Beta_Cl_(CA/creat) |  |  |  |  |  |  | -0.39 (0.12) | 31.6 |
| **Standard deviation of the random effects** | | | | | | | | |
| Omega_V | 0.4 (0.039) | 9.89 | 0.39 (0.04) | 10.1 | 0.39 (40.15) | 9.93 | 0.38 (0.036) | 9.57 |
| Omega_Cl | 0.5 (0.032) | 6.41 | 0.51 (0.034) | 6.57 | 0.49 (0.032) | 6.42 | 0.49 (0.031) | 6.38 |
| **Error model parameters** | | | | | | | | |
| a | 0.049 (0.022) | 45 | 0.065 (0.065) | 101 | 0.085 (0.085) | 55.1 | 0.07 (0.038) | 54.6 |
| b | 0.39 (0.013) | 3.37 | 0.4 (0.015) | 3.75 | 0.4 (0.015) | 3.77 | 0.4 (0.014) | 3.53 |


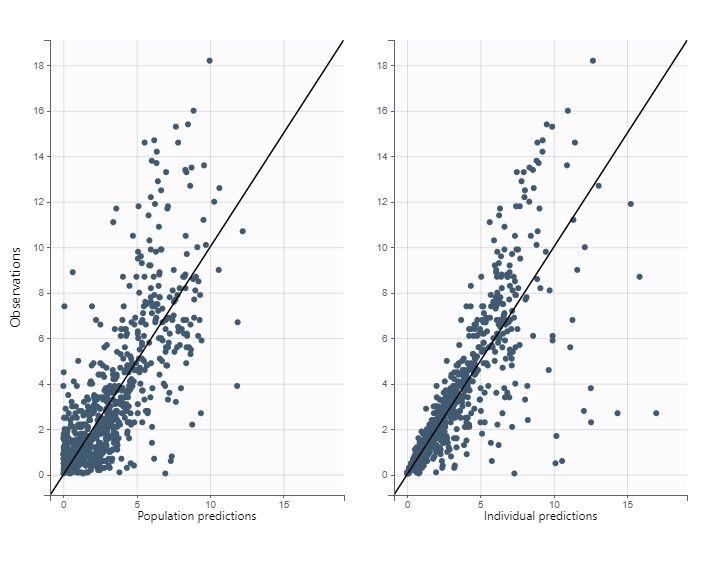


**Supplementary Figure 1** – Observed versus population and individual predicted concentrations for the final model.


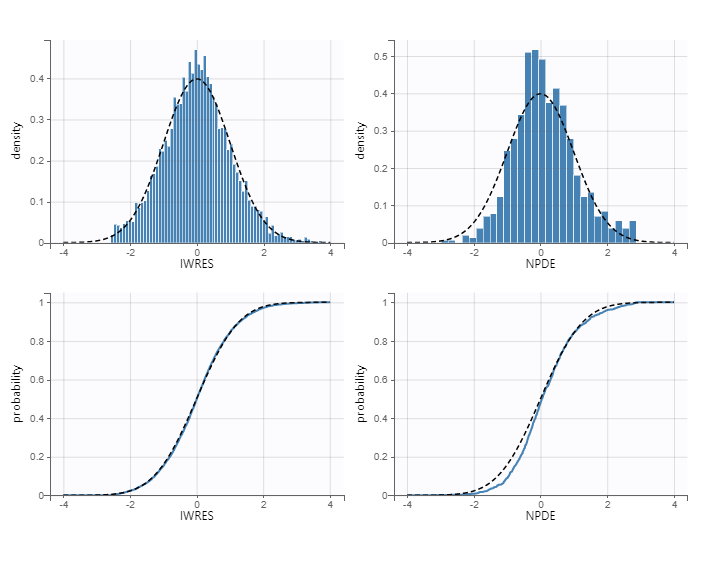


**Supplementary Figure 2** – Distribution of the individual weighted residuals and nonparametric distributional errors for the final model.
